# Supplementary material for: Aeromonas Species Diversity, Virulence Characteristics, and Antimicrobial Susceptibility Patterns in Village Freshwater Aquaculture Ponds in North India
Source: Antibiotics (Basel). 2025 Mar 12;14(3):294. doi: 10.3390/antibiotics14030294 (PMC11939274; doi:10.3390/antibiotics14030294)
Supplement: Supplementary file 1 [file antibiotics-14-00294-s001.zip › Supplementary Figure S1.pptx]

## Slide 1
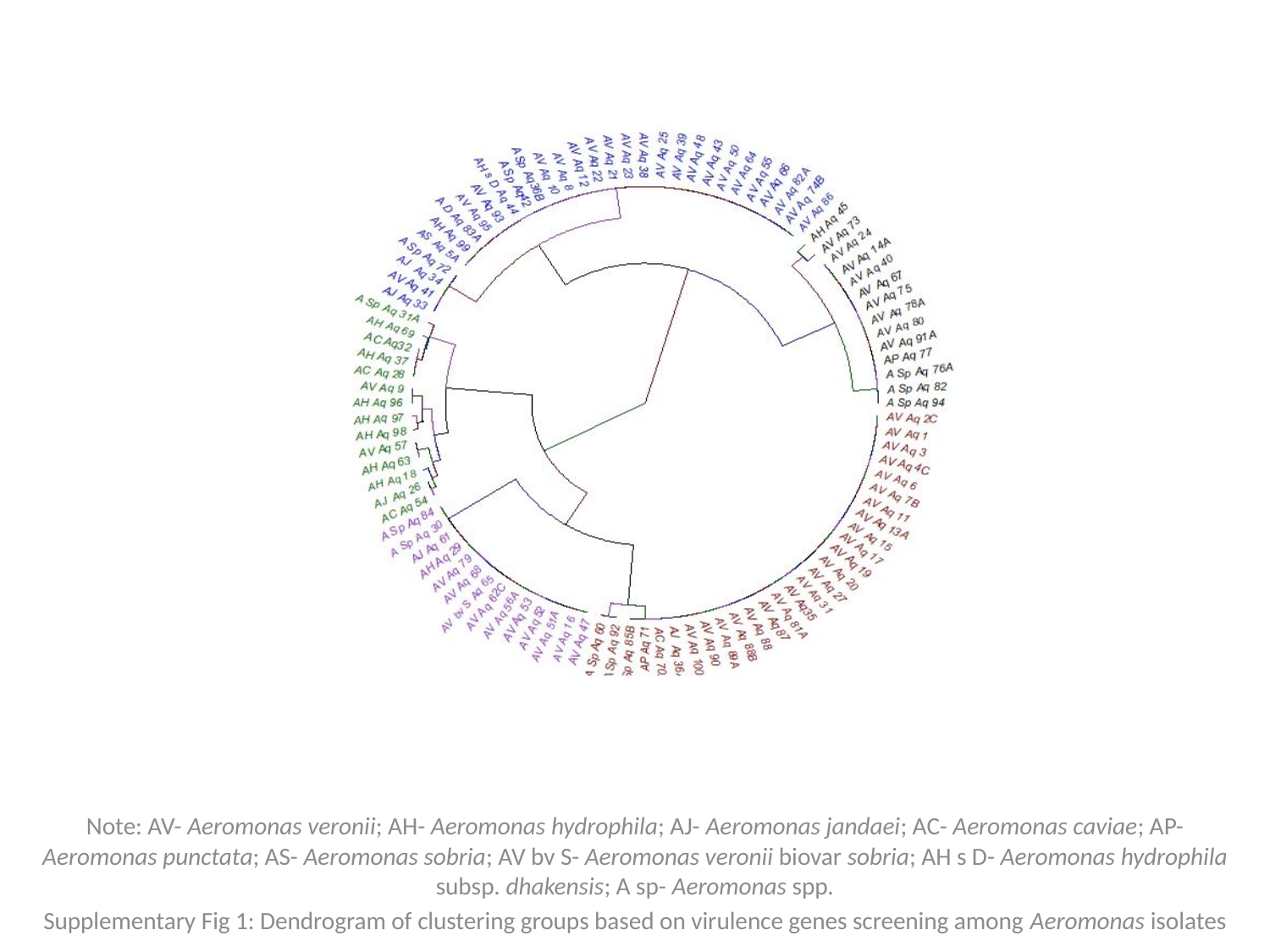

#
Note: AV- Aeromonas veronii; AH- Aeromonas hydrophila; AJ- Aeromonas jandaei; AC- Aeromonas caviae; AP- Aeromonas punctata; AS- Aeromonas sobria; AV bv S- Aeromonas veronii biovar sobria; AH s D- Aeromonas hydrophila subsp. dhakensis; A sp- Aeromonas spp.
Supplementary Fig 1: Dendrogram of clustering groups based on virulence genes screening among Aeromonas isolates
