# Supplementary material for: Aeromonas Species Diversity, Virulence Characteristics, and Antimicrobial Susceptibility Patterns in Village Freshwater Aquaculture Ponds in North India
Source: Antibiotics (Basel). 2025 Mar 12;14(3):294. doi: 10.3390/antibiotics14030294 (PMC11939274; doi:10.3390/antibiotics14030294)
Supplement: Supplementary file 1 [file antibiotics-14-00294-s001.zip › Supplementary Table S1.pdf]

Supplementary Table S1. Details of the primers used for screening virulence genes among *Aeromonas* isolates*

Primers selected to detect virulence genes				
Gene	Primer sequence (5'-3') F/R	Gene product	Ta (°C)	Size (bp)
*ahh*1	GCCGAGCGCCCAGAAGGTGAGTT/G AGCGGCTGGATGCGGTTGT	Extracellular hemolysin	60	130
*asa*1	TAAAGGGAAATAATGACGGCG/ GGCTGTAGGTATCGGTTTTCG	Hemolysin	56	249
act	AGAAGGTGACCACCAAGACA/ AACTGACATCGGCCTTGAACTC	Cytotoxic enterotoxin	56	232
ast	TCTCCATGCTTCCCTTCCACT/ GTGTAGGGATTGAAGAAGCCG	Heat-stable cytotonic enterotoxin	60	331
*asc*V	CTCGAACTGGAAGAGCAGAATG/ GAACATCTGGCTCTCCTTCTCCATG	Type III secretion system inner membrane component	60	577
eno	CGCCGACAACAACGTCGACATC/ CTTGATGGCAGCCAGAGTTTCG	Enolase	62	598
*aex*T	ATGCAGATTCAAGCAAACAC/ TTGCCGATCCACTCTTTGAT	ADP-ribosyating toxin	54	226

* Martino *et al* (2011).
