# Supplementary material for: Aeromonas Species Diversity, Virulence Characteristics, and Antimicrobial Susceptibility Patterns in Village Freshwater Aquaculture Ponds in North India
Source: Antibiotics (Basel). 2025 Mar 12;14(3):294. doi: 10.3390/antibiotics14030294 (PMC11939274; doi:10.3390/antibiotics14030294)
Supplement: Supplementary file 1 [file antibiotics-14-00294-s001.zip › Supplementary Table S2.pdf]

Supplementary Table S2. Biochemical characteristics of different *Aeromonas* species isolated from the fish culture ponds.

Biochemical test	*A. veronii*	*A. sobria*	*A. jandaei*	*A. dhakensis*	*A. caviae*	*A. hydrophila*	*Aeromonas* spp.	Total
	(n=59)	(n=6)	(n=4)	(n=7)	(n=1)	(n=2)	(n=18)	(n=97) *
	Percentage (%)							
**Gram stain**	-	-	-	-	-	-	-	100
**Oxidase**	+	+	+	+	+	+	+	100
**Catalase**	+	+	+	+	+	+	+	100
**Hugh Leifson Medium Test**								
**O/F**	72.9	100	75	0	85.7	83.3	77.8	75.3
**O/NF**	8.5	0	25	100	0	16.7	22.2	12.4
**NO/F**	8.5	0	0	0	0	0	0	5.2
**NR**	10.2	0	0	0	14.3	0	0	7.2
**Triple Sugar Iron Agar test**								
**Glucose(gas)**	72.9	50	75	0	28.6	66.7	77.8	69.1
**Glucose**	98.3	100	100	100	100	100	100	99
**Lactose/Sucrose**	37.3	50	50	0	14.3	50	33.3	36.1
**Citrate**	66.1	0	25	0	42.9	50	50	56.7
**Malonate**	10.2	0	0	0	0	0	5.6	7.2
**Indole**	89.8	100	100	100	71.4	100	77.8	87.6
**Motility**	100	100	100	100	100	100	100	100
**Urease**	0	0	0	0	0	0	0	0
**Haemolysis**								
**α**	35.6	50	100	100	42.9	16.7	27.8	37.1
**β**	59.3	50	0	0	57.1	66.7	72.2	58.8
**NH**	5.1	0	0	0	0	16.7	0	4.1

Note: (+): Present; (-): Absent; O/F: Oxidative/Fermentative; O/NF: Oxidative/Non-Fermentative; NO/F: Non-Oxidative/Fermentative; NR: No Reaction; NH; Non-Hemolytic; *Biochemical data includes 97 *Aeromonas* isolates, as confirmed by *gyr*B gene sequencing, excluding other species (n=3) such as *Stenotrophomonas* and *Psuedomonas*.
