# Supplementary material for: Aeromonas Species Diversity, Virulence Characteristics, and Antimicrobial Susceptibility Patterns in Village Freshwater Aquaculture Ponds in North India
Source: Antibiotics (Basel). 2025 Mar 12;14(3):294. doi: 10.3390/antibiotics14030294 (PMC11939274; doi:10.3390/antibiotics14030294)
Supplement: Supplementary file 1 [file antibiotics-14-00294-s001.zip › Supplementary Table S3.pdf]

Supplementary Table S3. *Aeromonas* isolates identification up to species level by *gyr*B sequences and GenBank Accession numbers.

Sr. No.	Genus and Species identified	Strain Number	GenBank Accession Number
1.	*Aeromonas veronii*	Aq1	ON501095
2.	*Aeromonas veronii*	Aq4C	ON501096
3.	*Aeromonas sobria*	Aq5A	ON501097
4.	*Aeromonas veronii*	Aq7B	ON501098
5.	*Aeromonas veronii*	Aq8	ON501099
6.	*Aeromonas veronii*	Aq13A	ON501100
7.	*Aeromonas veronii*	Aq14A	ON501101
8.	*Aeromonas veronii*	Aq22	ON501102
9.	*Aeromonas veronii*	Aq23	ON501103
10.	*Aeromonas hydrophila*	Aq29	ON501104
11.	*Aeromonasveronii*	Aq31	ON501105
12.	*Aeromonas caviae*	Aq31A	ON501106
13.	*Aeromonas caviae*	Aq32	ON501107
14.	*Aeromonas jandaei*	Aq34	ON501108
15.	*Aeromonas veronii*	Aq35	ON501109
16.	*Aeromonas veronii*	Aq36A	ON501110
17.	*Aeromonas hydrophila*	Aq37	ON501111
18.	*Aeromonas veronii*	Aq40	ON501112
19.	*Aeromonas hydrophila*	Aq45	ON501113
20.	*Aeromonas veronii*	Aq48	ON501114
21.	*Aeromonas veronii*	Aq51A	ON501115
22.	*Aeromonas veronii*	Aq53	ON501116
23.	*Aeromonas caviae*	Aq54	ON501117
24.	*Aeromonas veronii*	Aq57	ON501118
25.	*Aeromonas hydrophila*	Aq63	ON501119
26.	*Aeromonas veronii*	Aq64	ON501120
27.	*Aeromonas veronii*	Aq65	ON501121
28.	*Aeromonas veronii*	Aq68	ON501122
29.	*Aeromonas veronii*	Aq74B	ON501123
30.	*Aeromonas veronii*	Aq78A	ON501124
31.	*Aeromonas veronii*	Aq83A	ON501125
32.	*Aeromonas veronii*	Aq89A	ON501126
33.	*Aeromonas veronii*	Aq90	ON501127
34.	*Aeromonas veronii*	Aq2C	OP432055
35.	*Aeromonas veronii*	Aq6	OP432056
36.	*Aeromonas veronii*	Aq10	OP432057
37.	*Aeromonas veronii*	Aq12	OP432058
38.	*Aeromonas veronii*	Aq15	OP432059
39.	*Aeromonas veronii*	Aq16	OP432060
40.	*Aeromonas veronii*	Aq17	OP432061
41.	*Aeromonas hydrophila*	Aq18	OP432062
42.	*Aeromonas veronii*	Aq24	OP432063

43.	*Aeromonas jandaei*	Aq26	OP432064
44.	*Aeromonas veronii*	Aq27	OP432065
45.	*Aeromonas veronii*	Aq38	OP432066
46.	*Aeromonas veronii*	Aq42	OP432067
47.	*Aeromonas hydrophila*	Aq44	OP432068
48.	*Aeromonas veronii*	Aq50	OP432069
49.	*Aeromonas veronii*	Aq55	OP432070
50.	*Aeromonas veronii*	Aq56A	OP432071
51.	*Aeromonas veronii*	Aq66	OP432072
52.	*Aeromonas veronii*	Aq67	OP432073
53.	*Aeromonas caviae*	Aq70A	OP432074
54.	*Aeromonas veronii*	Aq73	OP432075
55.	*Aeromonas caviae*	Aq77	OP432076
56.	*Aeromonas veronii*	Aq81A	OP432077
57.	*Aeromonas veronii*	Aq86	OP432078
58.	*Aeromonas veronii*	Aq9	OR678517
59.	*Aeromonas veronii*	Aq11	OR678518
60.	*Aeromonas veronii*	Aq20	OR678519
61.	*Aeromonas veronii*	Aq39	OR678520
62.	*Aeromonas veronii*	Aq47	OR678521
63.	*Aeromonas veronii*	Aq52	OR678522
64.	*Aeromonas jandaei*	Aq61	OR678523
65.	*Aeromonas dhakensis*	Aq69	OR678524
66.	*Aeromonas caviae*	Aq71	OR678525
67.	*Aeromonas veronii*	Aq75	OR678526
68.	*Aeromonas veronii*	Aq80	OR678527
69.	*Aeromonas veronii*	Aq82A	OR678528
70.	*Aeromonas veronii*	Aq87	OR678529
71.	*Aeromonas veronii*	Aq3	OR678530
72.	*Aeromonas veronii*	Aq19	OR678531
73.	*Aeromonas sobria*	Aq25	OR678532
74.	*Aeromonas caviae*	Aq28	OR678533
75.	*Aeromonas jandaei*	Aq41	OR678534
76.	*Aeromonas veronii*	Aq62C	OR678535
77.	*Aeromonas veronii*	Aq72	OR678536
78.	*Aeromonas veronii*	Aq88	OR678537
79.	*Aeromonas veronii*	Aq88B	OR678538
