# Supplementary material for: Aeromonas Species Diversity, Virulence Characteristics, and Antimicrobial Susceptibility Patterns in Village Freshwater Aquaculture Ponds in North India
Source: Antibiotics (Basel). 2025 Mar 12;14(3):294. doi: 10.3390/antibiotics14030294 (PMC11939274; doi:10.3390/antibiotics14030294)
Supplement: Supplementary file 1 [file antibiotics-14-00294-s001.zip › Supplementary Table S4.pdf]

Supplementary Table S4. Antimicrobial resistance (AMR) assay for the different *Aeromonas* species isolates from the fish culture ponds.

	Response	FOX (30 mcg)	IPM (10 mcg)	CHL (25 mcg)	TET (30 mcg)	CPD (10 mcg)	AMC (30 mcg)	AMP (10 mcg)	CTX (30 mcg)	ATM (30 mcg)	AMK (30 mcg)	NAL (30 mcg)	CRO (30 mcg)	SXT (25 mcg)	CAZ (30 mcg)	NOR (10 mcg)
	Percentage (%)															
Total* (n=97)	S	67	93.8	92.8	94.8	89.7	95.9	0	80.4	89.7	92.8	57.7	91.8	90.7	87.6	100
	I	0	0	1.0	0	3.1	4.1	0	10.3	5.2	5.2	2.1	3.1	2.1	5.2	0
	R	33	6.2	6.2	5.2	7.2	0	100	9.3	5.2	2.1	40.2	5.2	7.2	7.2	0
A.veronii (n=59)	S	72.8	91.53	94.92	98.31	91.53	98.31	0	83.05	89.83	94.92	64.41	93.22	91.53	89.83	100
	I	0	0	0	0	5.08	2.69	0	8.47	5.08	3.39	0	5.08	3.39	5.08	0
	R	27.12	8.47	5.08	1.69	3.39	0	100	8.47	5.08	1.69	35.59	1.69	5.08	5.08	0
A.hydrophila (n=6)	S	16.67	100	66.67	50	66.67	100	0	66.67	83.33	100	33.33	100	83.33	66.67	100
	I	0	0	16.67	0	0	0	0	16.67	16.67	0	0	0	0	33.33	0
	R	83.33	0	16.67	50	33.33	0	100	16.67	0	0	66.67	0	16.67	0	0
A.jandaei (n=4)	S	100	100	100	100	50	100	0	50	100	100	50	100	100	100	100
	I	0	0	0	0	0	0	0	25	0	0	25	0	0	0	0
	R	0	0	0	0	50	0	100	25	0	0	25	0	0	0	0
A.caviae (n=7)	S	42.86	85.71	85.71	100	85.71	100	0	85.71	100	85.71	42.86	85.71	71.43	85.71	100
	I	0	0	0	0	0	0	0	0	0	0	0	0	0	0	0
	R	57.14	14.29	14.29	0	14.29	0	100	14.29	0	14.29	57.14	14.29	28.57	14.29	0
A.sobria (n=2)	S	100	100	50	100	100	100	0	50	50	50	100	100	100	100	100
	I	0	0	0	0	0	0	0	50	0	50	0	0	0	0	0
	R	0	0	50	0	0	0	100	0	50	0	0	0	0	0	0
A.dhakensis (n=1)	S	0	100	100	100	100	100	0	100	100	100	100	100	100	100	100
	I	0	0	0	0	0	0	0	0	0	0	0	0	0	0	0
	R	100	0	0	0	0	0	100	0	0	0	0	0	0	0	0
Aeromonas spp. (n=18)	S	67	93.8	92.8	94.8	89.7	95.9	0	80.4	89.7	92.8	57.7	91.8	90.7	87.6	100
	I	0	0	1.0	0	3.1	4.1	0	10.3	5.2	5.2	2.1	3.1	2.1	5.2	0
	R	33	6.2	6.2	5.2	7.2	0.0	100	9.3	5.2	2.1	40.2	5.2	7.2	7.2	0

Note: S- Sensitive; I- Intermediate; R- Resistant. *AMR data includes 97 *Aeromonas* isolates, as confirmed by *gyr*B gene sequencing, excluding other species (n=3) such as *Stenotrophomonas* and *Psuedomonas*.
