# Supplementary figures and images for: Aeromonas Species Diversity, Virulence Characteristics, and Antimicrobial Susceptibility Patterns in Village Freshwater Aquaculture Ponds in North India
Source: Antibiotics (Basel). 2025 Mar 12;14(3):294. doi: 10.3390/antibiotics14030294 (PMC11939274; doi:10.3390/antibiotics14030294)

## Slide 1
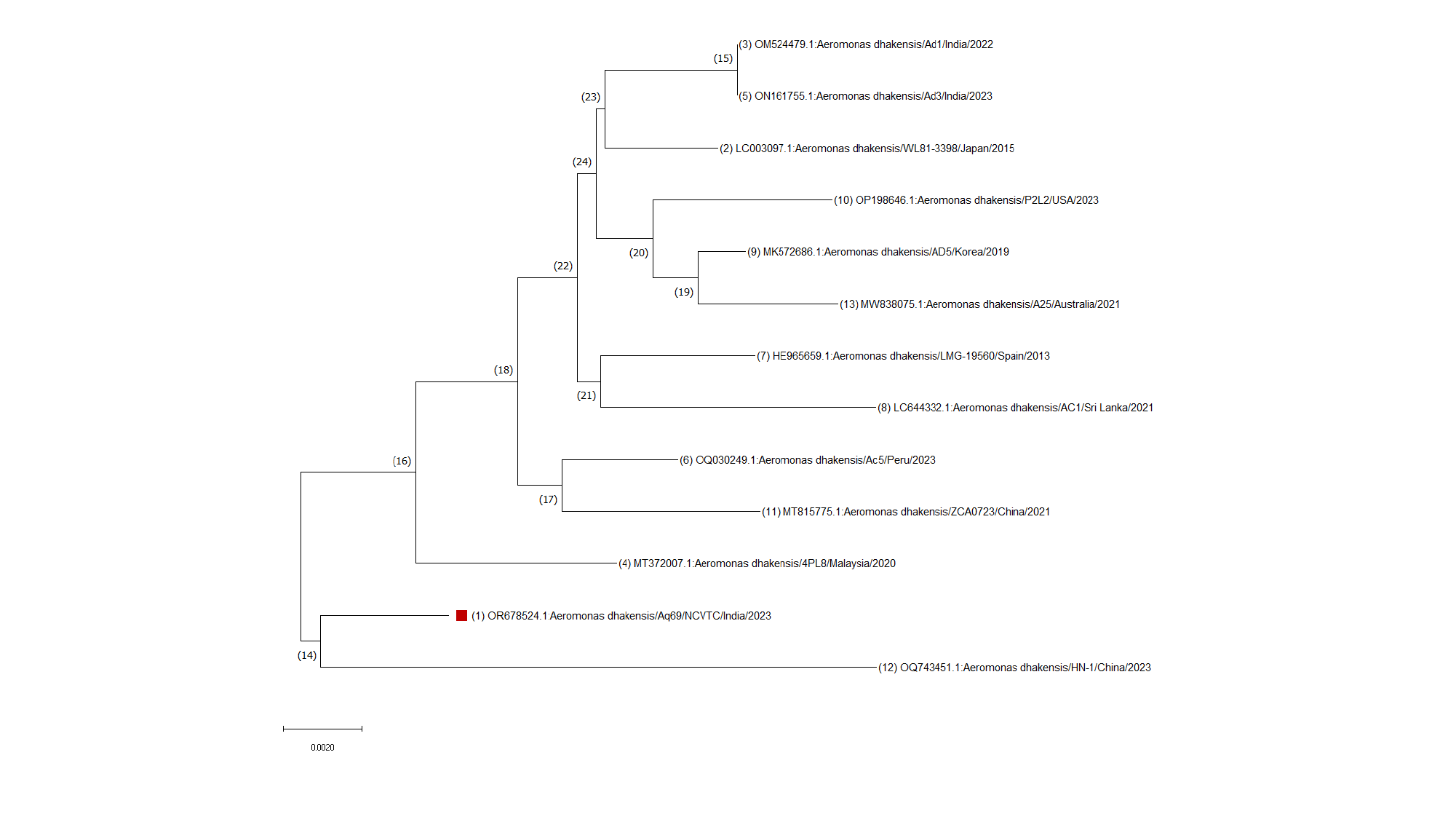

## Slide 2
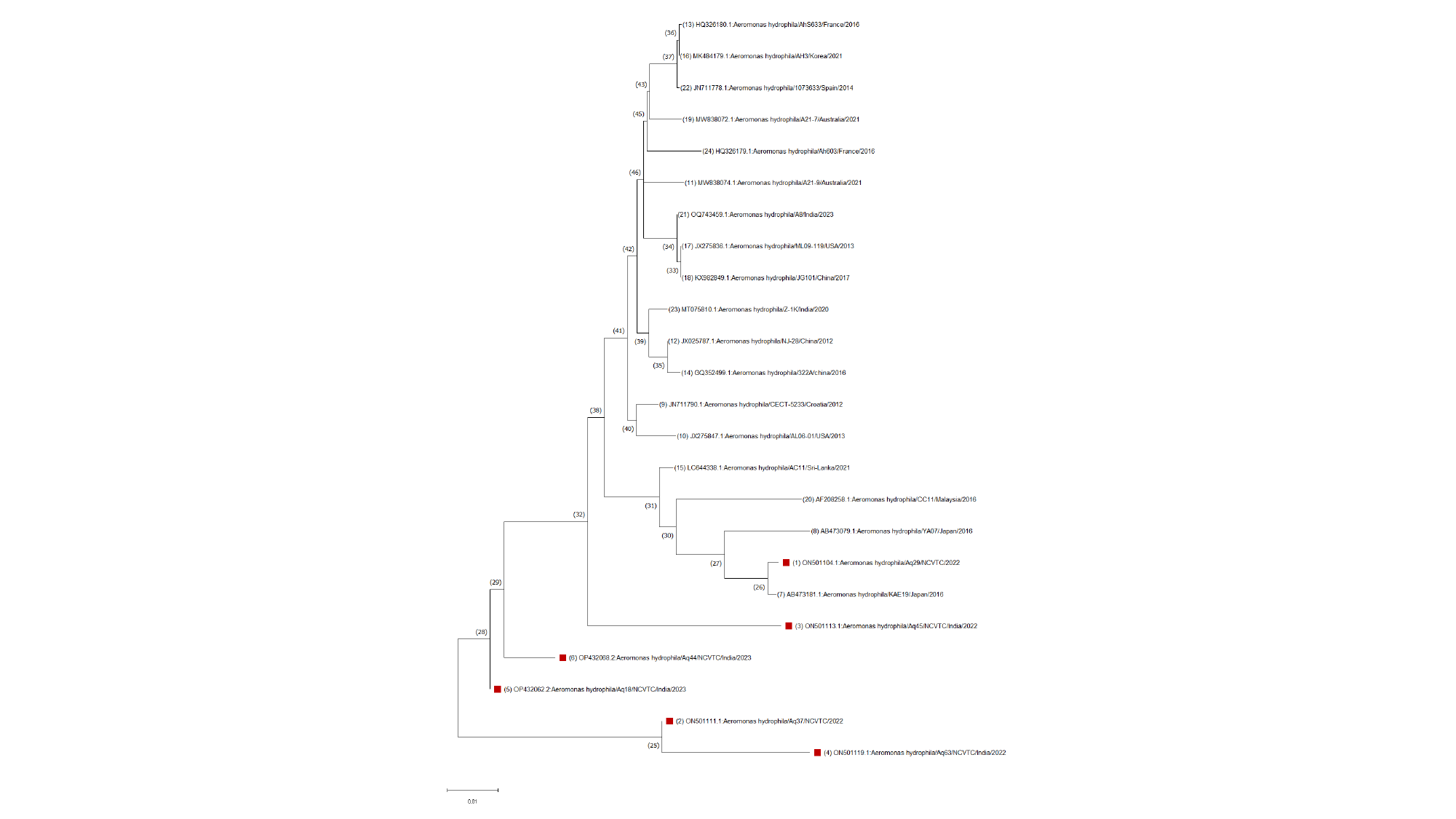

## Slide 3
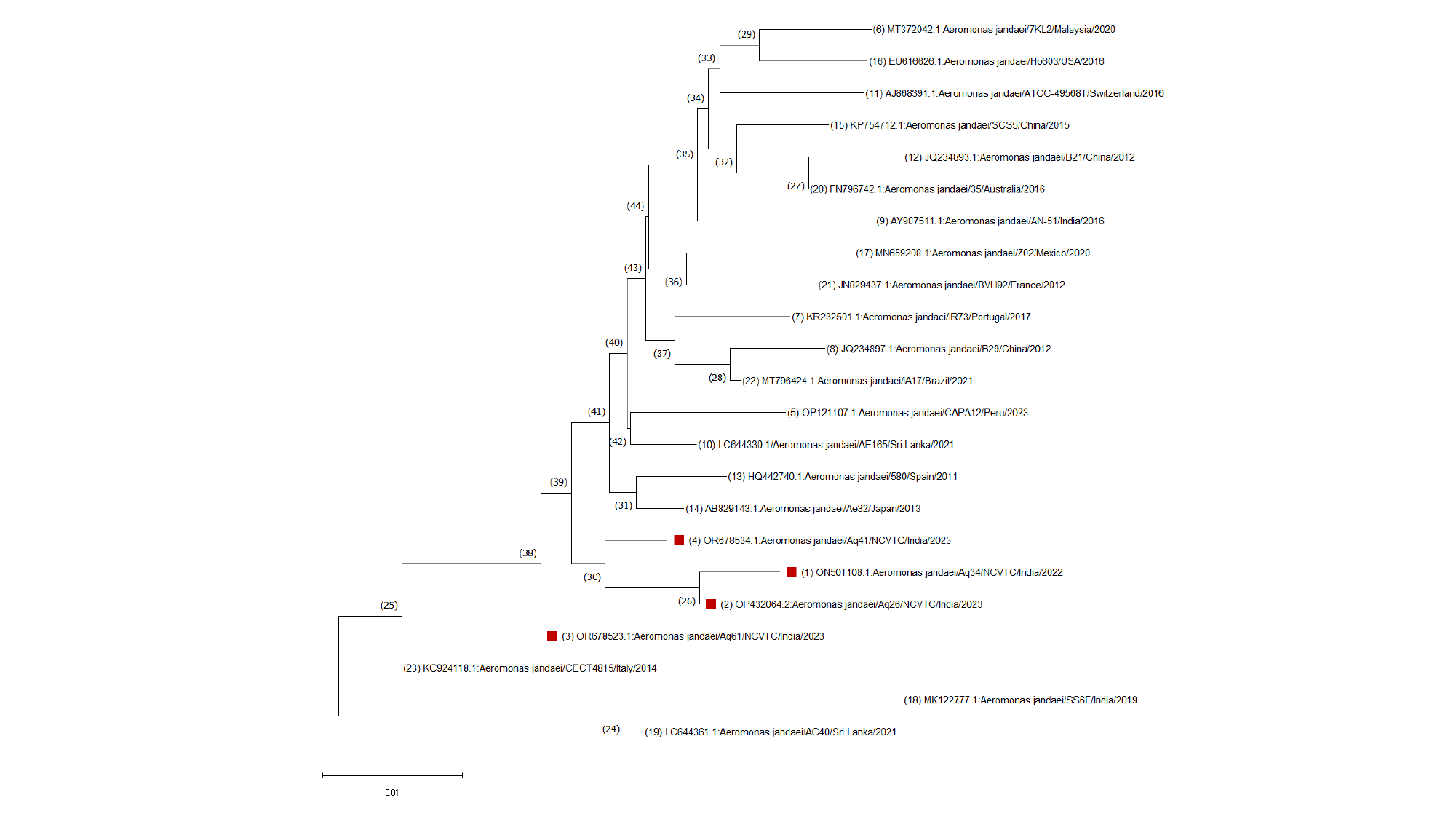

## Slide 4
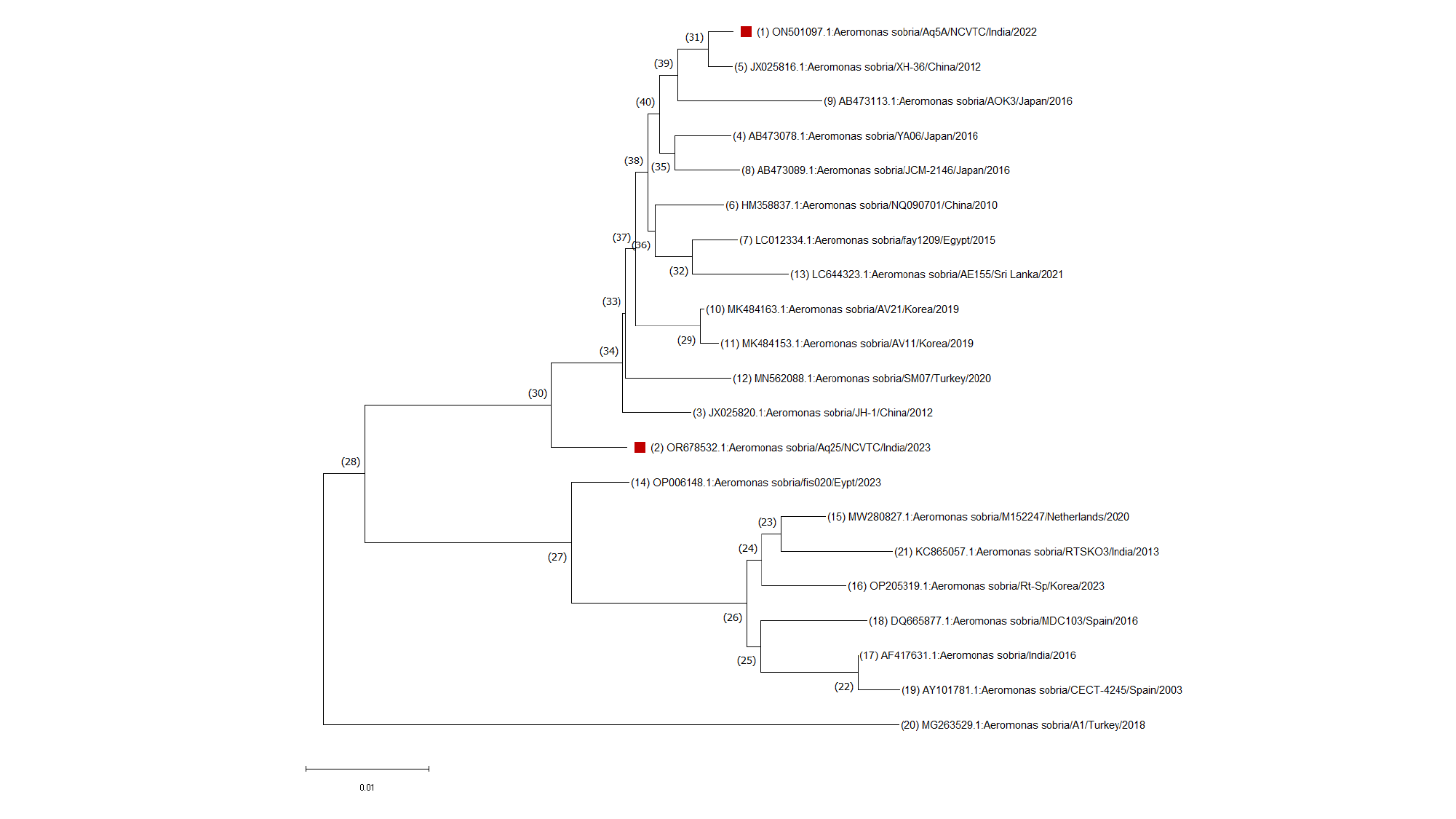

## Slide 5
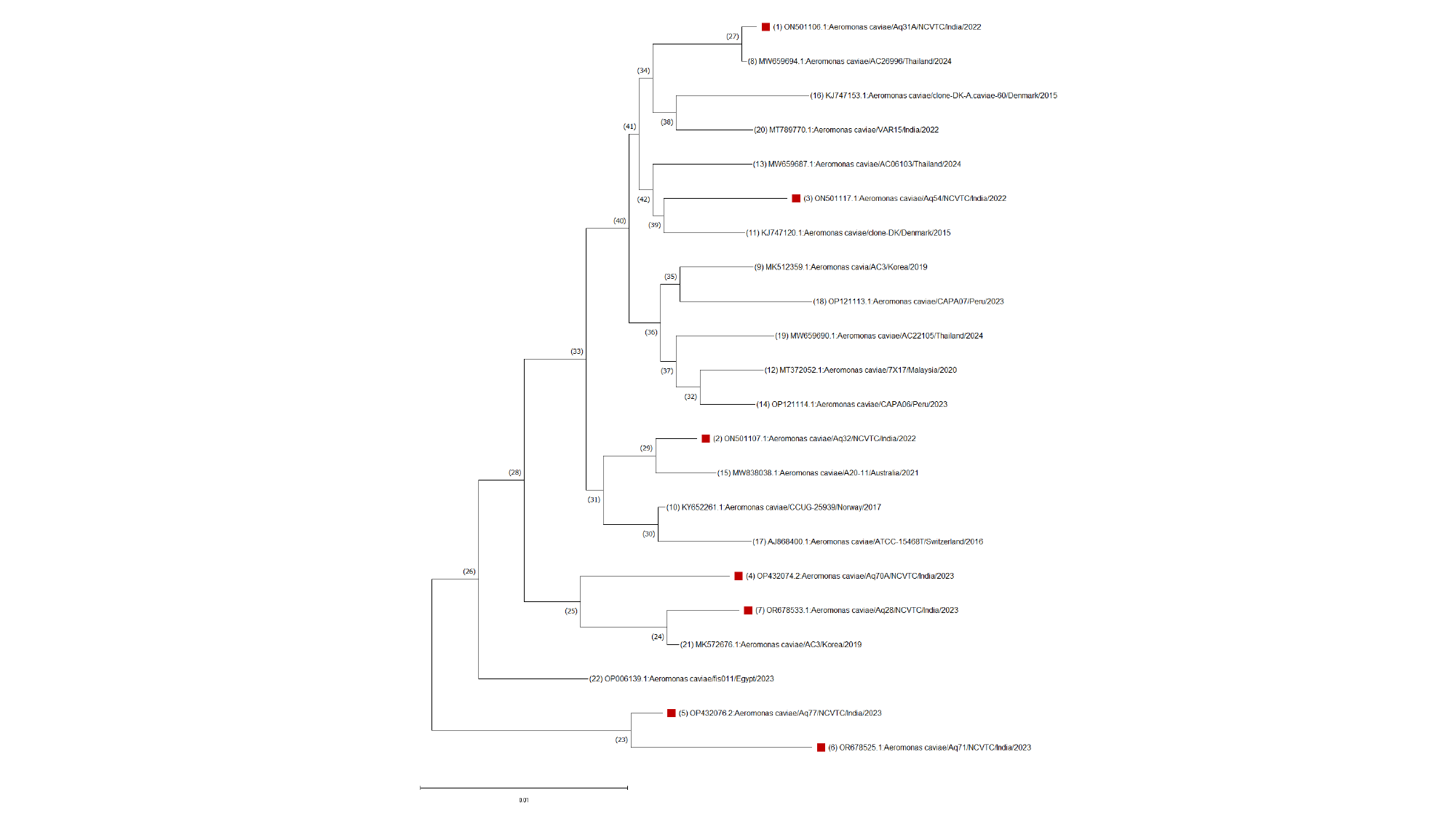

Supplement: Supplementary file 1 [file antibiotics-14-00294-s001.zip › Supplementary Figure S2.pptx]
